# Necdin modulates leukemia-initiating cell quiescence and chemotherapy response

**DOI:** 10.18632/oncotarget.20999

**Published:** 2017-09-18

**Authors:** Chonghua Yao, Michihiro Kobayashi, Sisi Chen, Sarah C. Nabinger, Rui Gao, Stephen Z. Liu, Takashi Asai, Yan Liu

**Affiliations:** ^1^ Department of Rheumatism, Shanghai Municipal Hospital of Traditional Chinese Medicine, Shanghai University of Traditional Chinese Medicine, Shanghai, China; ^2^ Department of Pediatrics, Herman B Wells Center for Pediatric Research, Indiana University School of Medicine, Indianapolis, IN, USA; ^3^ Department of Biochemistry and Molecular Biology, Indiana University School of Medicine, Indianapolis, IN, USA; ^4^ Sylvester Comprehensive Cancer Center, Miller School of Medicine, University of Miami, Miami, FL, USA

**Keywords:** Necdin, leukemia-initiating cells, quiescence, MLL-AF9, chemotherapy

## Abstract

Acute myeloid leukemia (AML) is a devastating illness which carries a very poor prognosis, with most patients living less than 18 months. Leukemia relapse may occur because current therapies eliminate proliferating leukemia cells but fail to eradicate quiescent leukemia-initiating cells (LICs) that can reinitiate the disease after a period of latency. While we demonstrated that p53 target gene Necdin maintains hematopoietic stem cell (HSC) quiescence, its roles in LIC quiescence and response to chemotherapy are unclear. In this study, we utilized two well-established murine models of human AML induced by MLL-AF9 or AML1-ETO9a to determine the role of Necdin in leukemogenesis. We found that loss of Necdin decreased the number of functional LICs and enhanced myeloid differentiation *in vivo*, leading to delayed development of leukemia induced by MLL-AF9. Importantly, Necdin null LICs expressing MLL-AF9 were less quiescent than wild-type LICs. Further, loss of Necdin enhanced the response of MLL-AF9^+^ leukemia cells to chemotherapy treatment, manifested by decreased viability and enhanced apoptosis. We observed decreased expression of *Bcl2* and increased expression of *p53* and its target gene *Bax* in Necdin null leukemia cells following chemotherapy treatment, indicating that p53-dependent apoptotic pathways may be activated in the absence of Necdin. In addition, we found that loss of Necdin decreased the engraftment of AML1-ETO9a^+^ hematopoietic stem and progenitor cells in transplantation assays. However, Necdin-deficiency did not affect the response of AML1-ETO9a^+^ hematopoietic cells to chemotherapy treatment. Thus, Necdin regulates leukemia-initiating cell quiescence and chemotherapy response in a context-dependent manner. Our findings suggest that pharmacological inhibition of Necdin may hold potential as a novel therapy for leukemia patients with MLL translocations.

## INTRODUCTION

Acute myeloid leukemia (AML) is the most common acute leukemia in adults. It usually occurs around age 60 with no identifiable cause and it carries a very poor prognosis, with most patients living less than 18 months [1]. Leukemia can be viewed as a malignancy initiated in a hematopoietic stem cell (HSC) or primitive progenitor cell that has maintained or acquired the capacity for self-renewal and is blocked in its ability to differentiate by the accumulation of a series of mutations and/or epigenetic changes [2–4]. The initial treatment of leukemia is designed to achieve a complete remission, meaning that the leukemic cells are not detectable in the bone marrow and normal blood formation has recovered. Leukemia relapse may occur because current therapies eliminate proliferating cells (that constitute the bulk of the disease) but fail to eradicate dormant leukemia-initiating cells (LICs) that can reinitiate the disease after a period of latency (the duration of remission) [1–2]. LICs, and in particular those that are in a dormant state, are resistant to chemotherapy or targeted therapies [5–7]. The development of new therapeutic approaches that can target LICs will have a profound impact on our ability to eradicate leukemia [8–10]. Unfortunately, little progress has been made in treating AML over the past 4 decades [1]. Clearly, new treatment strategies are urgently needed.

AML is characterized by recurrent chromosomal translocations, which generally target transcriptional regulatory genes, generating fusion proteins like MLL-AF9 and AML1-ETO [11–12]. MLL is a histone methyltransferase and MLL-AF9 is a 170 kDa fusion protein, generated by the t(9;11) [11]. MLL-AF9 is a frequently occurring MLL fusion oncogene typically associated with the FAB-M4 or M5 subtypes of human AML [11]. MLL associated leukemia accounts for the majority of infant leukemia, approximately 10% of adult *de novo* leukemia and approximately 33% of therapy related acute leukemia with a balanced chromosome translocation [11]. The presence of an MLL rearrangement generally confers a poor prognosis [1, 11]. MLL-AF9 is capable of transforming hematopoietic progenitor cells (HPCs) and HSCs, thus it can impart self-renewal to a non-self-renewing cell [13].

The t(8;21)(q22;q22) translocation is one of the most common genetic abnormalities in acute myeloid leukemia (AML), identified in 15% of all cases of AML, including 40–50% of FAB M2 subtype and rare cases of M0, M1 and M4 subtypes [12]. AML1-ETO is insufficient to cause acute leukemia by itself in human or mouse cells [14–15]. However, a truncated form of the AML1-ETO fusion protein (called AML1-ETO exon 9a) is sufficient to cause leukemia in mice, with a rather short latency [16–17]. AML1-ETO^+^ AML remains a significant clinical problem, with 30% of patients relapsing and long-term survival rates ranging between 30 and 60%, indicating the need for improved therapeutic approaches [18–19].

We are turning our attention to leukemia-initiating cells (LICs) to generate additional knowledge in order to develop therapeutic strategies that can eliminate the largely quiescent LICs and improve leukemia treatment. We have defined a critical role for p53 in regulating hematopoietic stem cell quiescence, and identified Necdin as a p53 target gene whose promoter binds and is transactivated by p53 [20–21]. Necdin is a growth suppressing protein first identified in post-mitotic neurons [22–23] and the gene encoding Necdin is one of several genes that are deleted in individuals with Prader-Willi syndrome [24]. Like the retinoblastoma protein, Necdin interacts with multiple cell cycle promoting proteins, such as simian virus 40 large T antigen, adenovirus E1A and the transcription factor E2F1 [25–27]. Necdin is highly expressed in long-term hematopoietic stem cells, and we have demonstrated that Necdin functions as a rheostat controlling HSC quiescence [21, 28]. Necdin null HSCs are more cycling and more easily exhausted, suggesting that Necdin is required for HSC maintenance [21].

Given that Necdin is essential for HSC quiescence and some patients with Prader-Willi syndrome develop AML [20–21, 29], we hypothesized that Necdin deficiency will stimulate quiescent LICs to enter the cell cycle and sensitize them to chemotherapy and improve leukemia treatment. To test this, we utilized two well-established mouse models of human AML, including MLL-AF9 and AML1-ETO9a, to determine the role of Necdin in LIC proliferation and chemotherapy response [13, 16]. We discovered that loss of Necdin decreased the quiescence of MLL-AF9^+^ LICs and sensitized leukemia cells expressing MLL-AF9 to chemotherapy treatment.

## RESULTS

### Necdin deficiency enhances the proliferation of hematopoietic progenitor cells expressing MLL-AF9

**Figure 1:**
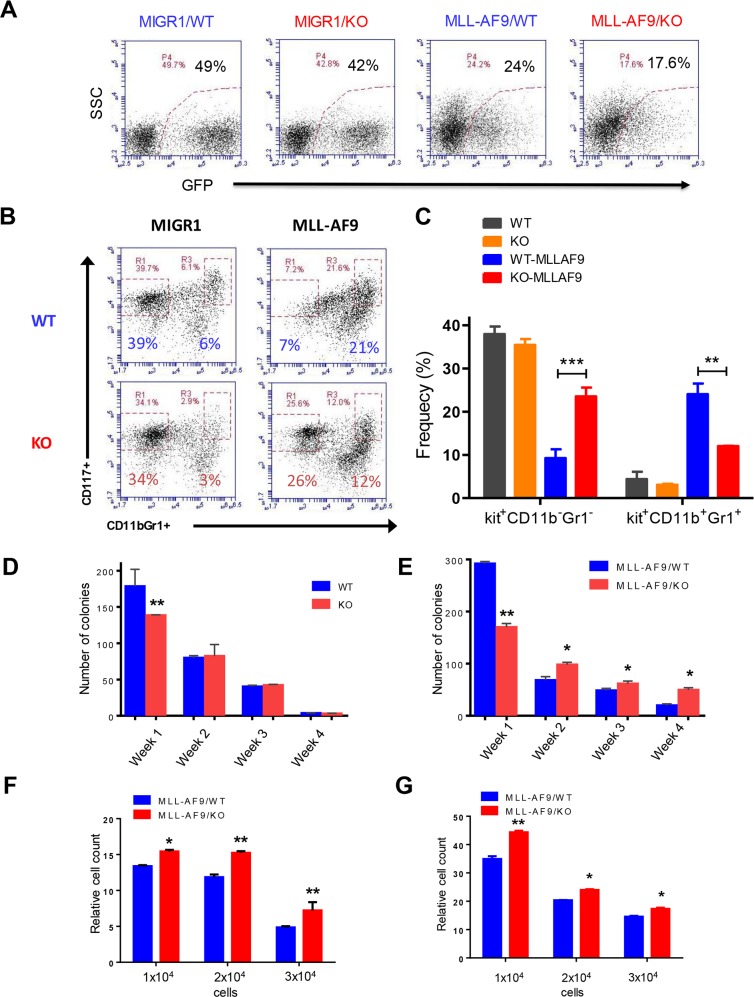
Necdin deficiency enhances the proliferation of hematopoietic progenitor cells expressing MLL-AF9 **(A)** Fetal liver cells isolated from wild-type (WT) or Necdin knock-out (KO) mice were transduced with retroviruses expressing GFP (MIGR1) or MLL-AF9. Representative flow cytometry plots show the frequency of transduced cells (GFP^+^) 72 hours following transduction. **(B)** Transduced wild type and Necdin null fetal liver cells (GFP^+^) were cultured in serum free medium in the presence of cytokines for seven days. The frequency of hematopoietic stem and progenitor cells was determined by flow cytometry analysis. Representative flow cytometry plots show the frequency of Kit^+^CD11b^−^Gr1^−^ and Kit^+^CD11b^+^Gr1^+^ cells at 7 days in liquid culture. **(C)** The frequency of Kit^+^CD11b^−^Gr1^−^ and Kit^+^CD11b^+^Gr1^+^ cells in the liquid culture (^**^p<0.01, ^***^p<0.001, n=2). **(D)** Serial replating studies. CFUs were quantified by methylcellulose culture using WT and Necdin null fetal liver cells. The methylcellulose cultures were serially replated, weekly, for 4 weeks. Mean values (± SD) were shown (^**^p<0.01, n=3). **(E)** Necdin null fetal liver cells expressing MLL-AF9 show enhanced replating potential compared to WT cells (^*^p<0.05, ^**^p<0.01, n=3). **(F)** and **(G)** Liquid culture of WT and Necdin null fetal liver cells expressing MLL-AF9. 48 (F) and 72 (G) hours later, cell proliferation was determined by cell counting. Cell growth was presented relative to the number of input cells in each group, set as 1 (^*^p<0.05, ^**^p<0.01, n=3).

We utilized a mouse model of human AML induced by the MLL-AF9 oncogene to determine the role of Necdin in the initiation and progression of AML [13]. We infected wild type and Necdin null fetal liver cells, which contain hematopoietic stem and progenitor cells (HSPCs), with retroviruses expressing GFP or MLL-AF9. Robust expression of the GFP was seen 72h post-infection (Figure 1A). We cultured transduced cells (GFP^+^) in serum free medium in the presence of cytokines for seven days and then examined the frequency of HSPCs. We found that loss of Necdin increased the frequency of Kit^+^CD11b^−^Gr1^−^ cells and decreased the frequency of Kit^+^CD11b^+^Gr1^+^ cells (Figure 1B and 1C). Given that leukemia-initiating cells or leukemia stem cells (LSCs) in murine model of MLL-AF9^+^ AML are Kit^+^CD11b^+^Gr1^+^ cells [13, 30], our finding suggests that Necdin-deficiency may decrease the number of LICs in MLL-AF9-induced leukemia.

In the serial replating assay, wild-type fetal liver cells cannot be replated more than three times (Figure 1D). While Necdin null HSPCs show decreased colony formation in week 1, the replating potentials of Necdin null cells and wild-type cells are comparable (Figure 1D). We then performed serial replating assays using wild type and Necdin null fetal liver cells expressing MLL-AF9. While Necdin null cells show decreased colony formation in week one compared to wild-type cells, they show enhanced colony formation potential compared to wild-type cells in following weeks (Figure 1E). To determine the effect of Necdin deficiency on HSPC proliferation, we cultured wild type and Necdin null fetal liver cells expressing MLL-AF9 in medium containing hematopoietic cytokines. 48 and 72 hours later, we counted cell number using flow cytometry and found that expression of MLL-AF9 enhanced the proliferation of Necdin null HSPCs compared to wild type HSPCs (Figures 1F and 1G).

### Necdin deficiency delays the progression of leukemia-induced by MLL-AF9

**Figure 2:**
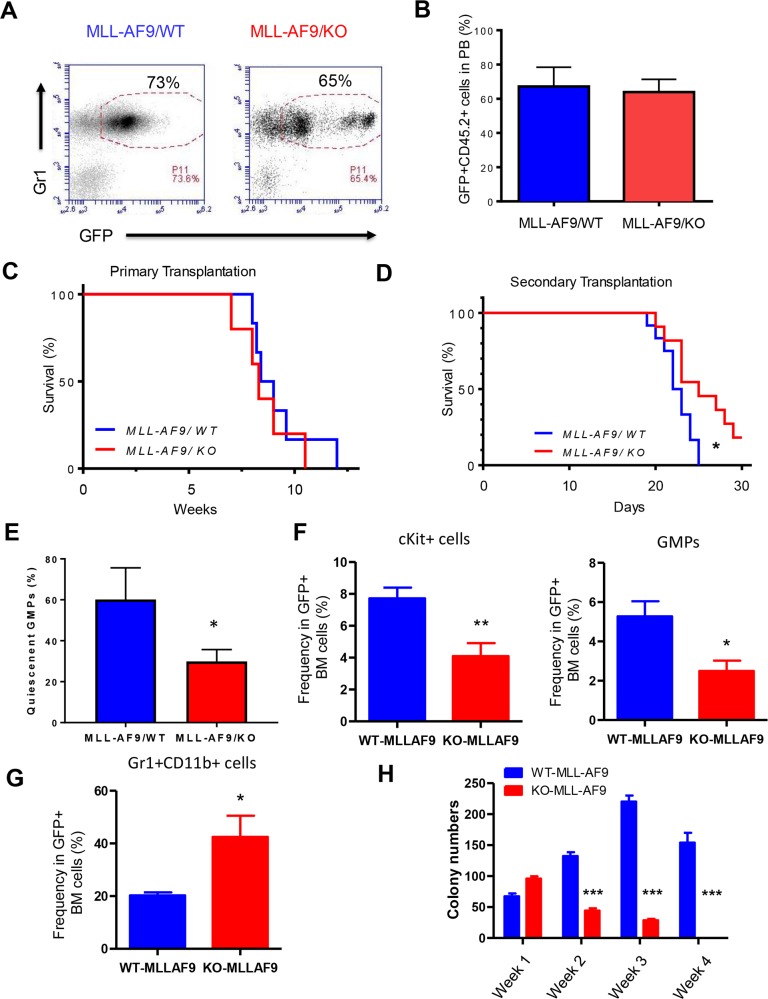
Necdin deficiency delays the progression of leukemia-induced by MLL-AF9 **(A)** Primary transplantation of fetal liver cells expressing MLL-AF9. Representative flow cytometry plots show the frequency of GFP^+^Gr1^+^ cells in the peripheral blood of recipient mice 4 weeks following transplantation. **(B)** The frequency of donor-derived cells (CD45.2^+^GFP^+^) in the peripheral blood (PB) of recipient mice was determined by flow cytometry analysis (p=0.2, n=5). **(C)** Survival curve of animals transplanted with wild type or Necdin null fetal liver cells expressing MLL-AF9 (p=0.2, n=5). **(D)** Bone marrow cells isolated from primary recipient mice were transplanted into lethally irradiated recipient mice. Survival curve of recipient mice transplanted with WT or Necdin null leukemia cells expressing MLL-AF9 (^*^p<0.05, n=7). **(E)** The quiescence of GMPs in the BM of leukemia mice were determined by Ki67 and DAPI staining. Quiescent cells are defined as Ki67^−^ cells (^*^p<0.05, n=3). **(F)** The frequency of Kit^+^ cells and GMPs in the BM of leukemia mice was determined by flow cytometry analysis (^*^p<0.05, ^**^p<0.01, n=5). **(G)** The frequency of myeloid cells (Gr1^+^CD11b^+^) in the BM of leukemia mice was determined by flow cytometry analysis (^*^p<0.05, n=5). **(H)** Necdin null GMPs expressing MLL-AF9 show decreased replating potential compared to wild type leukemia cells (^***^p<0.001, n=3).

To determine the role of Necdin in MLL-AF9-induced leukemia, we transplanted 100,000 wild type or Necdin null HSPCs expressing MLL-AF9 cells (CD45.2^+^ GFP^+^) into lethally irradiated recipient mice (B6.SJL mice, CD45.1^+^) together with 100,000 normal competitor cells (CD45.1^+^). We monitored leukemia progression in recipient mice by checking GFP^+^ leukemic cells in the peripheral blood every 4 weeks. We observed rapid expansion of GFP^+^ leukemia cells in recipient mice repopulated with both wild -type and Necdin null HSPCs expressing MLL-AF9 at 4 weeks following transplantation (Figures 2A and 2B). We observed several population of Gr1^+^ cells with different GFP intensity in the peripheral blood of recipient mice repopulated with Necdin null HSPCs expressing MLL-AF9 (Figure 2A), suggesting that Necdin may affect the differentiation of LICs expressing MLL-AF9.

In the bone marrow transduction and transplantation model of MLL-AF9, all recipient mice developed AML, with a median latency of 84.5 days [13]. We utilized fetal liver cells and obtained similar results. There is no apparent effect of Necdin deficiency on the survival of recipient mice and all recipient mice transplanted with wild type or Necdin null mice died 11 weeks following transplantation (Figure 2C), suggesting that Necdin is dispensable for the initiation and/or progression of leukemia induced by MLL-AF9 in primary transplantation assays. We transplanted 3 × 10^6^ GFP^+^ MLL-AF9^+^ bone marrow cells (CD45.2^+^) isolated from the primary recipient mice into lethally irradiated recipient mice (B6.SJL, CD45.1^+^). We monitored leukemia development in recipient mice. While it takes 10 weeks for MLL-AF9 to induce leukemia in the primary recipient mice (Figure 2C), it only takes 3 weeks for these leukemia cells to develop leukemia in secondary recipients (Figure 2D). Importantly, we found that Necdin-deficiency significantly delayed leukemia onset (Figure 2D), suggesting that Necdin is important for the progression of leukemia induced by MLL-AF9 in transplantation assays.

Given that Necdin maintains HSC quiescence [20–21] and GMPs (Lin^−^Sca1^−^IL7R^−^Kit^+^FcγRII/III^high^CD34^high^) are leukemia-initiating cells (LICs) in MLL-AF9 induced leukemia [13], we examined the impact of Necdin deficiency on the quiescent state of GMPs using Ki67 and DAPI staining. We found that loss of Necdin significantly decreased the number of quiescent GMPs (Ki67^−^) in the bone marrow of leukemia mice (Figure 2E), suggesting that Necdin maintains the quiescence of leukemia-initiating cells expressing MLL-AF9.

To further investigate the mechanisms by which Necdin deficiency delays leukemia progression, we examined the frequency of Kit^+^ cells, GMPs, and Gr1^+^CD11b^+^ cells in the bone marrow of leukemia mice. We found that loss of Necdin decreased the frequency of Kit^+^ cells and GMPs in the bone marrow (Figure 2F), suggesting that the number of LICs was decreased in the bone marrow of leukemia mice in the absence of Necdin. Further, we observed decreased number of mature myeloid cells (Gr1^+^CD11b^+^) in the bone marrow of leukemia mice repopulated with Necdin null cells (Figure 2G), indicating that Necdin deficiency promotes myeloid differentiation. To determine the functional impact of Necdin deficiency on LICs, we performed serial replating assays. While Necdin null LICs (GMPs) generated more colonies than wild type cells in Week 1, these cells show decreased colony formation capability compared to wild type leukemia cells in following weeks (Figure 2H), demonstrating that Necdin deficiency impairs LIC function *in vitro*.

### Loss of Necdin sensitizes leukemia cells expressing MLL-AF9 to chemotherapy

**Figure 3:**
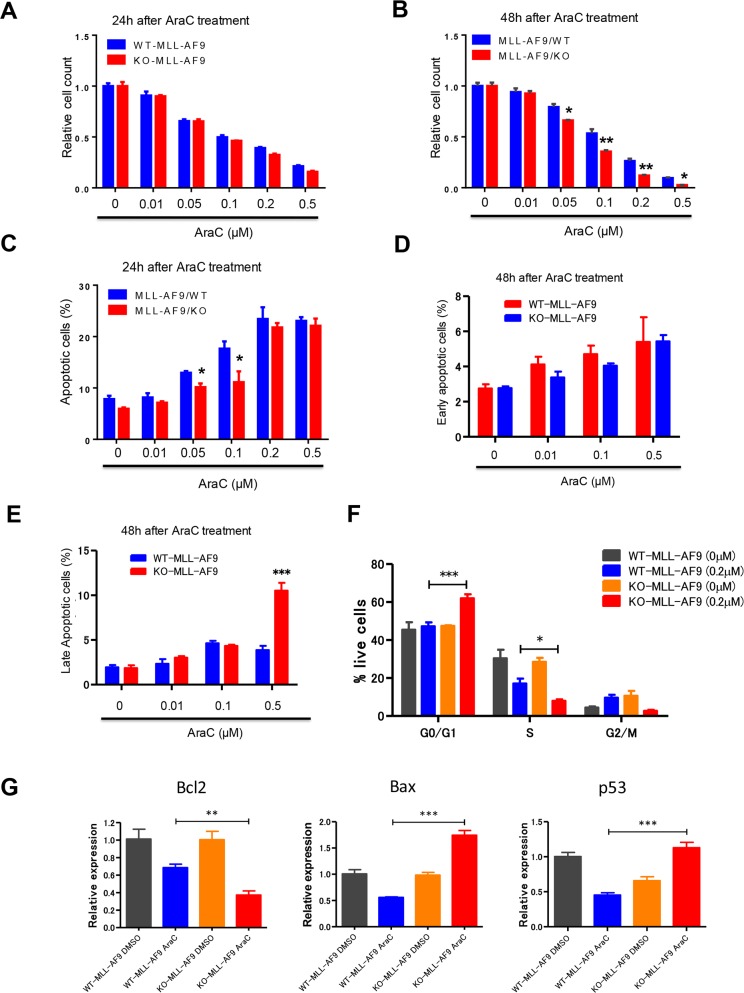
Necdin null leukemia cells expressing MLL-AF9 are sensitive to chemotherapy treatment **(A)** and **(B)** WT and Necdin null leukemia cells expressing MLL-AF9 were treated with DMSO or different concentrations of chemotherapy drug cytarabine (AraC). 24 (A) and 48 (B) hours after AraC treatment, the viability of treated leukemia cells was measured by cell counting (^*^p<0.05, ^**^p<0.01, n=3). **(C)** WT and Necdin null leukemia cells expressing MLL-AF9 were treated with DMSO or different concentrations of AraC. 24 hours after AraC treatment, the frequency of total apoptotic cells (Annexin V^+^) was determined by flow cytometry analysis (^*^p<0.05, n=3). **(D)** and **(E)** WT and Necdin null leukemia cells expressing MLL-AF9 were treated with DMSO or different concentrations of AraC. 48 hours after AraC treatment, the frequency of early apoptotic cells (Annexin V^+^PI^−^) and late apoptotic cells (Annexin V^+^PI^+^) was determined by flow cytometry analysis (^***^p<0.001, n=3). **(F)** Wild type and Necdin null leukemia cells were treated with DMSO or AraC (0.2 μM). 24 hours later, cell cycle status of leukemia cells was determined by flow cytometry analysis (^*^p<0.05, ^***^p<0.001, n=3). **(G)** Wild type and Necdin null leukemia cells were treated with DMSO or AraC. 6 hours later, the expression of *Bcl2, Bax*, and *p53* in leukemia cells was determined by quantitative real-time PCR analysis (^**^p<0.01, ^***^p<0.001, n=3).

Given that loss of Necdin decreased the quiescence of GMPs expressing MLL-AF9 (Figure 2E), we predicted that Necdin null leukemia cells expressing MLL-AF9 would be sensitive to chemotherapy. That was indeed the case. We treated wild type and Necdin null leukemia cells expressing MLL-AF9 with DMSO or different concentrations of chemotherapy drug cytarabine (AraC) and monitored cell viability by cell counting. While 24 hour AraC treatment did not affect the viability of leukemia cells expressing MLL-AF9 (Figure 3A), we found that Necdin null leukemia cells expressing MLL-AF9 were sensitive to extended AraC treatment (48 hour) in a dosage-dependent manner, manifested by decreased viability (Figure 3B). While the LC50 for AraC on wild type leukemia cells is 0.16 μM, the LC50 for AraC on Necdin null leukemia cells expressing MLL-AF9 is 0.09 μM. We then examined the impact of AraC treatment on leukemia cell survival. The number of total apoptotic cells was comparable between two groups following 24 hour AraC treatment (Figure 3C). While loss of Necdin did not affect the early apoptosis of leukemia cells expressing MLL-AF9 following 48 hour AraC treatment (Figure 3D), the number of late apoptotic cells (Annexin V^+^/PI^+^) was significantly increased in Necdin null group compared with wild type group following high concentration of AraC (0.5 μM) treatment (Figure 3E). We then performed cell cycle analysis of leukemia cells treated with DMSO or AraC (0.2 μM). While AraC treatment did not affect the cell cycle status of wild type leukemia cells expressing MLL-AF9, AraC treatment increased the number of Necdin null leukemia cells in the G0/G1 phase of the cell cycle and decreased the number of Necdin null leukemia cells in the S phase of the cell cycle (Figure 3F).

To understand the molecular basis of enhanced apoptosis and cell cycle arrest seen in the Necdin null leukemia cells following AraC treatment, we examined the expression of genes that regulate apoptosis and cell cycle, including *Bcl2*, *Bax* and *p53*, in leukemia cells treated with DMSO or AraC. We observed decreased levels of *Bcl2* and increased levels of *p53* and its target gene *Bax* in Necdin null leukemia cells compared to wild type leukemia cells following AraC treatment (Figure 3G). While Bcl2 inhibits apoptosis, Bax promotes p53-dependent apoptosis following genotoxic stress [31–32]. These findings suggest that Necdin deficiency may activate the p53-dependent apoptotic pathways in MLL-AF9^+^ leukemia cells following AraC treatment.

### Necdin deficiency decreases the proliferation of hematopoietic progenitor cells expressing AML1-ETO9a

**Figure 4:**
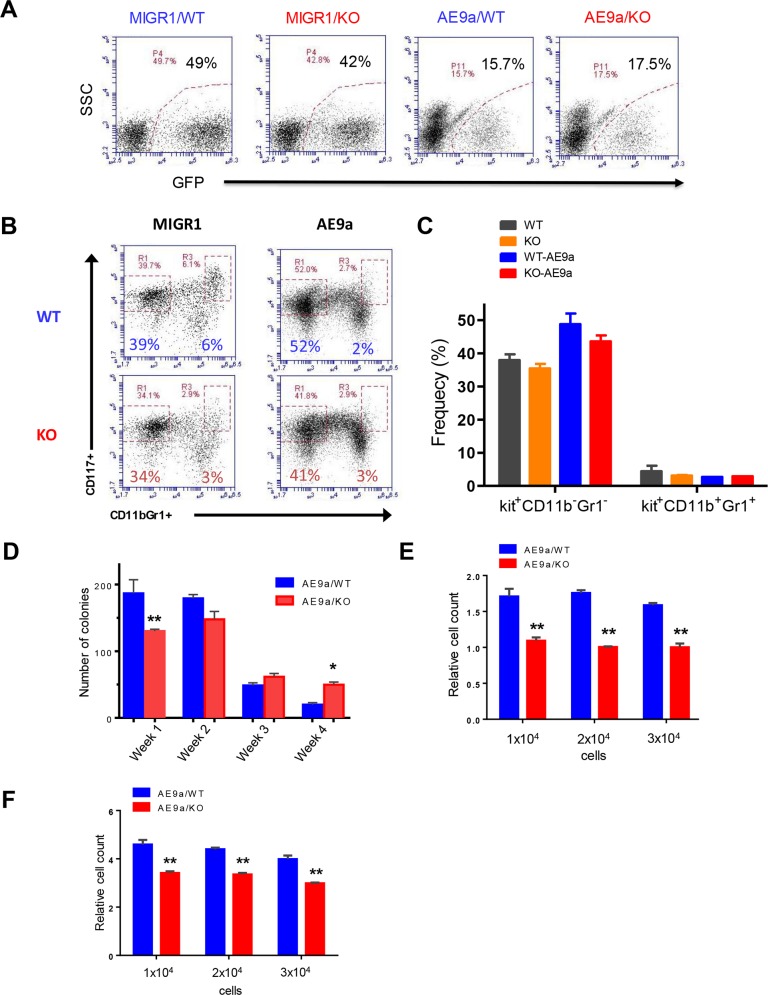
Necdin deficiency decreases the proliferation of hematopoietic progenitor cells expressing AML1-ETO9a **(A)** Fetal liver cells isolated from wild-type (WT) or Necdin knock-out (KO) mice were transduced with retroviruses expressing GFP (MIGR1) or AML1-ETO9a. Representative flow cytometry plots show the frequency of transduced cells (GFP^+^) 72 hours following transduction. **(B)** Transduced wild type and Necdin null fetal liver cells (GFP^+^) were cultured in serum free medium in the presence of cytokines for seven days. The frequency of hematopoietic stem and progenitor cells was determined by flow cytometry analysis. Representative flow cytometry plots show the frequency of Kit^+^CD11b^−^Gr1^−^ and Kit^+^CD11b^+^Gr1^+^ cells at 7 days in liquid culture. **(C)** The frequency of Kit^+^CD11b^−^Gr1^−^ and Kit^+^CD11b^+^Gr1^+^ cells in the liquid culture (p<0.2, n=2). **(D)** Necdin null fetal liver cells expressing AML1-ETO9a show enhanced replating potential compared to WT cells (^*^p<0.05, ^**^p<0.01, n=3). **(E)** Liquid culture of WT and Necdin null fetal liver cells expressing AML1-ETO9a. Cell proliferation at 48 hours was determined by cell counting. Cell growth was presented relative to the number of input cells in each group, set as 1 (^**^p<0.01, n=3). **(F)** Liquid culture of WT and Necdin null fetal liver cells expressing AML1-ETO9a. Cell proliferation at 72 hours was determined by cell counting. Cell growth was presented relative to the number of input cells in each group, set as 1 (^**^p<0.01, n=3).

In addition to MLL-AF9-induced leukemia, we also utilized a mouse model of human AML induced by the AML-ETO9a oncogene to determine the role of Necdin in leuekmia initiation and progression [16]. We infected wild type and Necdin null HSPCs with retroviruses expressing GFP or AML1-ETO9a. Robust expression of GFP was seen 72h post-infection (Figure 4A). We then cultured transduced cells (GFP^+^) in serum free medium in the presence of cytokines for seven days and then examined the frequency of HSPCs. We found comparable numbers of Kit^+^CD11b^−^Gr1^−^ and Kit^+^CD11b^+^Gr1^+^ cells in both wild type and Necdin null cell cultures (Figures 4B and 4C). We performed serial replating assays using wild type and Necdin null HSPCs expressing AML-ETO9a. While Necdin null HSPCs show decreased colony formation in week one compared to that of the WT cells, these cells show enhanced colony formation than that of the wild type cells by week 4 (Figure 4D). We then examine the effect of Necdin deficiency on HSPC proliferation and found that Necdin null HSPCs expressing AML1-ETO9a show decreased proliferation compared to wild type HSPCs (Figures 4E and 4F), indicating that Necdin is important for the proliferation of HSPCs expressing AML-ETO9a.

### Necdin deficiency decreases the repopulating potential of hematopoietic stem and progenitor cells expressing AML1-ETO9a

**Figure 5:**
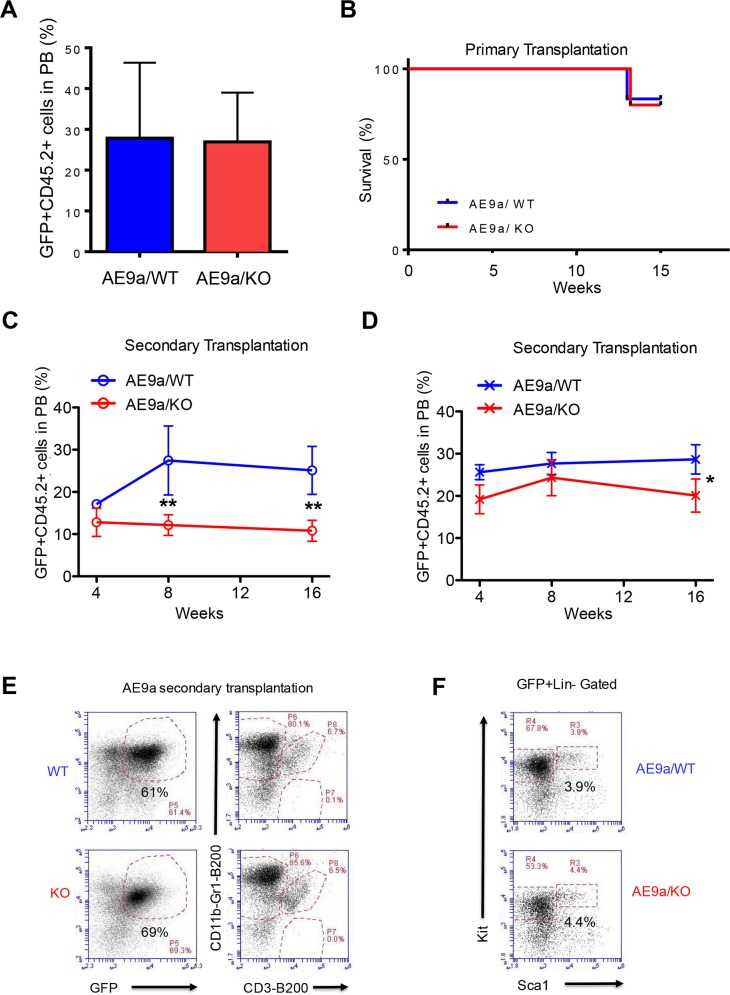
Necdin deficiency decreases the repopulating potential of hematopoietic stem and progenitor cells expressing AML1-ETO9a **(A)** Primary transplantation of fetal liver cells expressing AML1-ETO9a. The frequency of donor-derived cells (CD45.2^+^GFP^+^) in the peripheral blood (PB) of recipient mice at 8 weeks following transplantation was determined by flow cytometry analysis (p=0.2, n=5-6). **(B)** Survival curve of animals transplanted with WT or Necdin null fetal liver cells expressing AML1-ETO9a (P=0.2, n=5-6). **(C)** and **(D)** Secondary transplantation assays using 3 × 10^5^ (C) or 3 ×10^6^ (D) bone marrow cells from mice repopulated with WT or Necdin null fetal liver cell expressing AML1-ETO9a. The frequency of donor-derived cells (CD45.2^+^GFP^+^) in peripheral blood was measured by flow cytometry analysis every four weeks for 16 weeks. (^*^p<0.05, ^**^p<0.01, n=5). (E) The frequency of donor-derived (CD45.2^+^GFP^+^) myeloid cells (CD11b^+^Gr1^+^), B cells (B220^+^), and T cells (CD3^+^) in the bone marrow of secondary recipient mice at 16 weeks following transplantation was determined by flow cytometry analysis. **(F)** The frequency of donor-derived (CD45.2^+^GFP^+^) Lin^−^Sca1^−^Kit^+^ and Lin^−^Sca1^+^Kit^+^ cells in the bone marrow of secondary recipient mice at 16 weeks following transplantation was determined by flow cytometry analysis.

To determine the role of Necdin in AML-ETO9a -induced leukemia, we transplanted 100,000 wild type or Necdin null HSPCs expressing AML-ETO9a (CD45.2^+^GFP^+^) into lethally irradiated recipient mice (B6.SJL mice, CD45.1^+^) together with 100,000 normal competitor cells (CD45.1^+^). In primary transplantation, we observed no difference in the frequency of donor-derived GFP^+^ cells in mice repopulated with wild type or Necdin null HSPCs expressing AML-ETO9a at 8 weeks following transplantation (Figure 5A). There is no apparent effect of Necdin deficiency on the survival of recipient mice as most recipient mice transplanted with wild type or Necdin null mice are still alive 14 weeks following transplantation (Figure 5B), suggesting the Necdin may be dispensable for AML1-ETO9a-mediated leukemia initiation.

To determine the impact of Necdin deficiency on the repopulating potential of hematopoietic stem and progenitor cells expressing AML1-ETO9a, we sacrificed the primary recipient mice 16 weeks following transplantation and harvested GFP^+^ bone marrow cells for secondary transplantation. We performed Limiting-dilution transplantation assays by transplanting 3 × 10^5^ or 3 × 10^6^ AML1-ETO^+^ bone marrow cells (CD45.2^+^ GFP^+^) isolated from the primary recipient mice into lethally irradiated recipient mice (B6.SJL, CD45.1). We monitored engraftment of GFP^+^ hematopoietic cells in peripheral blood by flow cytometry every 4 weeks after transplantation. We found that Necdin null bone marrow cells show decreased engraftment using both concentrations of donor cells 16 weeks post transplantation (Figures 5C and 5D), suggesting that loss of Necdin decreases the repopulating potential of hematopoietic stem and progenitor cells expressing AML1-ETO9a.

To determine the impact of Necdin deficiency on hematopoietic stem and progenitor cells *in vivo*, we analyzed the bone marrow of secondary recipient mice repopulated with wild type or Necdin null bone marrow cells expressing AML1-ETO9a. We observed comparable number of donor-derived (CD45.2^+^GFP^+^) myeloid cells (CD11b^+^Gr1^+^), B cells (B220^+^), and T cells (CD3^+^) in the bone marrow of recipient mice from both the wild type and the Necdin null groups (Figure 5E), indicating that Necdin deficiency does not affect terminal differentiation of HSPCs expressing AML1-ETO9a. In addition, we found comparable numbers of donor-derived (CD45.2^+^GFP^+^) hematopoietic progenitor cells (Lin^−^Sca1^−^Kit^+^ and Lin^−^Sca1^+^Kit^+^) in the bone marrow of recipient mice from both groups, suggesting that Necdin deficiency does not alter the frequency HSPCs expressing AML1-ETO9a *in vivo* (Figure 5F).

### The impact of AraC treatment on hematopoietic cells expressing AML1-ETO9a

**Figure 6:**
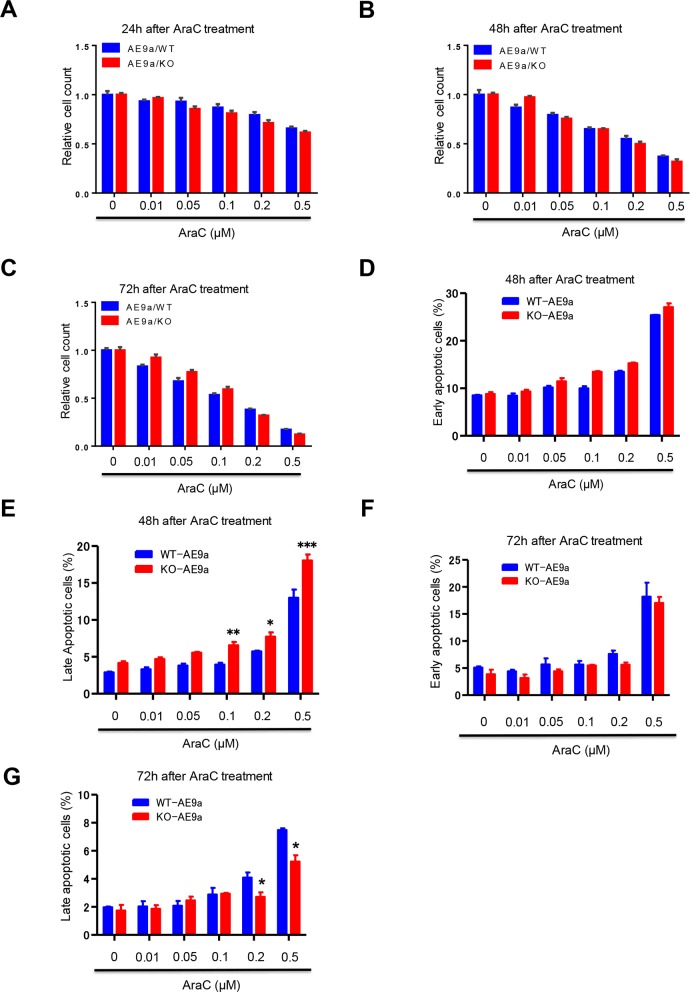
The impact of AraC treatment on hematopoietic cells expressing AML1-ETO9a **(A)**, **(B)**, and **(C)** WT and Necdin null hematopoietic cells expressing AML1-ETO9a were treated with DMSO or different concentrations of chemotherapy drug cytarabine (AraC). 24 (A), 48 (B), and 72 (C) hours after AraC treatment, the viability of treated hematopoietic cells was measured by cell counting (n=3). **(D)** and **(E)** WT and Necdin null hematopoietic cells expressing AML1-ETO9a were treated with DMSO or different concentrations of AraC. 48 hours after AraC treatment, the frequency of early apoptotic cells (Annexin V^+^PI^−^) and late apoptotic cells (Annexin V^+^PI^+^) was determined by flow cytometry analysis (^*^p<0.05, ^**^p<0.01, ^***^p<0.001, n=3). **(F)** and **(G)** WT and Necdin null hematopoietic cells expressing AML1-ETO9a were treated with DMSO or different concentrations of AraC. 72 hours after AraC treatment, the frequency of early apoptotic cells (Annexin V^+^PI^−^) and late apoptotic cells (Annexin V^+^PI^+^) was determined by flow cytometry analysis (^*^p<0.05, n=3).

Given that loss of Necdin decreases the proliferation of hematopoietic progenitor cells expressing AML1-ETO9a (Figure 4E and 4F), we predicted that Necdin-deficient hematopoietic cells expressing AML1-ETO9a would not be sensitive to chemotherapy. We isolated wild type and Necdin null hematopoietic cells from the secondary recipient mice repopulated with hematopoietic cells expressing AML1-ETO9a. We treated wild type and Necdin null cells with DMSO or different concentrations of chemotherapy drug cytarabine (AraC) and monitored cell viability by cell counting. We found that the viability of wild type and Necdin null hematopoietic cells expressing AML1-ETO9a were comparable following AraC treatment (Figures 6A, 6B and 6C). The LC50 for AraC on wild type cells is 0.15 μM and the LC50 for AraC on Necdin-deficient cells expressing AML1-ETO9a is 0.14 μM. We then examined the impact of AraC treatment on the survival of hematopoietic cells expressing AML1-ETO9a. While loss of Necdin did not affect the number of early apoptotic (Annexin V^+^/PI^−^) leukemia cells expressing AML1-ETO9a (Figure 6D), the number of late apoptotic cells (Annexin V^+^/PI^+^) was significantly increased in Necdin null group compared with wild type group following high concentration of AraC treatment (Figure 6E). We also examined the apoptosis of hematopoietic cells 72 hours after AraC treatment. Although the number of early apoptotic cells was comparable in both groups (Figure 6F), the number of late apoptotic cells was decreased in the Necdin null group compared to wild type group (Figure 6G).

## DISCUSSION

Many cancers seem to depend on a small population of ‘cancer stem cells’ for their continued growth and propagation [2]. The leukemia-initiating cell (LIC) or leukemia stem cell (LSC) was the first such cell to be described [2–3]. Leukemia-initiating cells (LICs), particularly those that are in a quiescent state, are resistant to chemotherapy or targeted therapies [5–7]. The LIC is likely to be the most crucial target in the treatment of leukemia, and a thorough understanding of its biology - particularly how the LIC differs from the HSC - might allow it to be selectively targeted, improving therapeutic outcome [2, 8]. However, the critical regulators of LIC quiescence are largely unknown [9–10]. Thus, deciphering the molecular mechanisms controlling LIC quiescence is essential for developing therapeutic strategies that can eliminate quiescent LICs.

Quiescence (G0) is a physiological state occupied by “resting” cells that have exited the cell cycle [9]. It was generally thought that the HSC pool turn over every few weeks [33–34]. However, recent findings suggest that there are dormant HSCs and activated HSCs in laboratory animals [35]. Dormant HSCs are efficiently activated in response to bone marrow injury or G-CSF stimulation. However, once the steady state is reestablished, the activated HSCs return to dormancy, suggesting that HSCs reversibly switch from dormancy to self-renewal during homeostasis and repair [35]. Both HSC-intrinsic regulators and bone marrow niche factors contribute to HSC quiescence [9]. Cell cycle regulators, such as PTEN, Rb, PML, and MEF, regulate hematopoietic stem cell quiescence [36–40]. Stem cell niche signals, such as Angiopoietin/Tie2 signaling, promote HSC quiescence [41].

We demonstrated that p53 plays a critical role in regulating HSC quiescence and identified Necdin as an important p53 target gene in HSCs [20]. To determine the role of Necdin in hematopoiesis, we analyzed the hematopoietic compartment of a strain of Necdin-null mice that die perinatally and have features resembling those seen in the human Prader-Willi Syndrome [42]. While Necdin functions like p53 to maintain HSC quiescence during steady state, Necdin opposes p53-dependent apoptosis under conditions of genotoxic stress [21]. Despite being a p53 target, Necdin appears to both mimic and antagonize p53 function in hematopoietic stem and progenitor cells [20–21]. Recently, down regulation of p53 target genes, including *Necdin* and *Gfi-1*, in hematopoietic stem and progenitor cells, has been shown to promote ENU-induced leukemogenesis [43]. While some patients with Prader-Willi Syndrome develop AML [29], the role of Necdin in leukemogenesis is largely unknown. We utilized two well-established murine models of human AML induced by MLL-AF9 and AML1-ETO9a to determine the role of Necdin in leukemia development and discovered that Necdin controls leukemia-initiating cell quiescence and chemotherapy response in a context-dependent manner.

Murine fetal liver mainly consists of hematopoietic stem and progenitor cells that are highly proliferative [44]. To determine the impact of Necdin on hematopoietic progenitor cell proliferation, we introduced oncogenic fusion proteins, including MLL-AF9 and AML1-ETO9a, into wild type and Necdin null fetal liver cells and performed proliferation assays. While loss of Necdin enhanced the proliferation of fetal liver cells expressing MLL-AF9 (Figures 1F and 1G), Necdin deficiency decreased the proliferation of fetal liver cells expressing AML1-ETO9a (Figures 4E and 4F). Thus, Necdin appears to play distinct roles in regulating the proliferation of hematopoietic progenitor cells expressing different oncogenic fusion proteins. The leukemia-initiating cells in MLL-AF9-induced leukemia are GMPs [13]. We found that Necdin null GMPs expressing MLL-AF9 were less quiescent than wild-type GMPs (Figure 2E), suggesting that Necdin maintains the quiescence of LICs expressing MLL-AF9. Whether Necdin contributes to the quiescence of LICs expressing AML1-ETO9a is not known. Based upon the effect of Necdin deficiency on normal hematopoietic progenitor cells expressing AML1-ETO9a (Figures 4E and 4F), we predict that loss of Necdin may enhance the quiescence of AML1-ETO9a^+^ LICs.

In the bone marrow transduction and transplantation model of MLL-AF9, all recipient mice developed AML, with a median latency of 84.5 days [13]. To determine the role of Necdin in MLL-AF9-induced leukemia, we introduced MLL-AF9 into wild type and Necdin null fetal livers cells and performed transplantation assays. In primary transplantation assays, the survival rate between wild type and Necdin null groups was comparable (Figure 2C); However, Necdin deficiency significantly delayed the development of leukemia induced by MLL-AF9 in secondary transplantation assays (Figure 2D). These findings suggest that Necdin may be important for the progression but not the initiation of leukemia induced by MLL-AF9. To understand the mechanisms by which Necdin deficiency delays leukemia onset, we characterized the behavior of GMPs in the bone marrow of the secondary recipient mice. We found that loss of Necdin decreased the number of GMPs in the bone marrow of recipient mice (Figure 2F). Further, Necdin null GMPs show decreased replating capability compared to wild-type GMPs (Figure 2H). These findings indicate that Necdin deficiency decreases both the number and the function of GMPs expressing MLL-AF9. In addition, we found increased number of myeloid cells in the bone marrow of recipient mice repopulated with Necdin null bone marrow cells compared to that of the wild-type cells (Figure 2G). Thus, the delayed leukemia development seen in the recipient mice repopulated with Necdin null bone marrow cells expressing MLL-AF9 may be due to decreased number of GMPs as well as enhanced myeloid differentiation.

While AML1-ETO is insufficient to cause acute leukemia by itself in human or mouse cells [14–15], AML1-ETO9a fusion protein is sufficient to cause leukemia in mice [16–17]. To determine the role of Necdin in AML1-ETO9a-induced leukemia, we introduced AML1-ETO9a into wild type and Necdin null fetal livers cells and performed transplantation assays. Sixteen weeks following primary transplantation assays, all recipient mice were still alive (Figure 4B). Given that hematopoietic progenitor cells expressing AML1-ETO9a show enhanced replating potential *in vitro* (Figure 4D), we predicted that loss of Necdin would increase the repopulating potential of bone marrow cells expressing AML1-ETO9a *in vivo*. To test this, we isolated bone marrow cells from primary recipient mice repopulated with wild type and Necdin null fetal liver cells and performed secondary transplantation assays with limiting dilution of donor bone marrow cells. Surprisingly, loss of Necdin decreased the number of donor-derived cells in the peripheral blood of secondary recipient mice 16 weeks post transplantation (Figures 5C and 5D), suggesting that Necdin deficiency may decrease the repopulating potential of AML1-ETO9a^+^ hematopoietic stem and progenitor cells in transplantation assays. We only monitored the survival of secondary recipient mice for 16 weeks and all mice were alive at the time of sacrifice. Given that Necdin deficiency did not affect the number of hematopoietic stem and progenitor cells and their differentiation in the bone marrow (Figures 5E and 5F), we predict that the survival rate would be comparable between wild type and Necdin null groups in secondary transplantation.

Despite advances in the treatment of acute myeloid leukemia (AML), relapse and drug resistance frequently occur [1]. As Necdin null HSCs are less quiescent than wild-type HSCs and wild type mice repopulated with Necdin null HSCs show enhanced sensitivity to weekly 5-FU treatments or sub-lethal doses of irradiation [21], targeting Necdin may provide a therapeutic approach to eliminating quiescent leukemia-initiating cells. Given that quiescent LICs are resistant to chemotherapy treatment [5–9], we predicted that decreased GMP quiescence would sensitize Necdin-deficient leukemia cells expressing MLL-AF9 to AraC treatment. Indeed, we found that Necdin deficiency enhanced the response of leukemia cells expressing MLL-AF9 to chemotherapy treatment, manifested by decreased viability and enhanced apoptosis (Figures 3B and 3E). To understand the mechanisms by which Necdin controls chemo-sensitivity, we examined the expression of p53 and apoptosis related genes in leukemia cells treated with DMSO or AraC. We found that the mRNA levels of *p53* and its target gene *Bax* were increased in Necdin null leukemia cells expressing MLL-AF9 compared to wild-type leukemia cells (Figure 3G). However, the expression of apoptosis regulatory gene *Bcl2* was decreased in Necdin null leukemia cells compared to wild-type leukemia cells (Figure 3G). Necdin physically interacts with p53 and suppresses p53-dependent apoptosis in neurons and hematopoietic cells [21, 25, and 45]. These data suggest that p53-dependent apoptotic pathways may be activated in Necdin null leukemia cells expressing MLL-AF9 following AraC treatment. Interestingly, AraC treatment induced cell cycle arrest in Necdin null leukemia cells expressing MLL-AF9 (Figure 3F). The cell cycle arrest may be due to increased expression of p53 in Necdin null cells following AraC treatment (Figure 3G). Thus, Necdin deficiency may sensitize leukemia cells expressing MLL-AF9 to AraC treatment through decreasing LIC quiescence and inducing p53-dependent apoptosis and cell cycle arrest.

In contrary to the effect of Necdin deficiency on the response of leukemia cells expressing MLL-AF9 to AraC treatment, loss of Necdin did not affect the viability of leukemia cells expressing AML1-ETO9a following chemotherapy treatment (Figures 6A, 6B and 6C). While Necdin null leukemia cells expressing AML1-ETO9a show enhanced apoptosis at 48 hours after high concentration of AraC treatment (Figure 6E), these cells had decreased apoptosis at 72 hours after AraC treatment (Figure 6G). Given that Necdin null leukemia cells expressing either MLL-AF9 or AML1-ETO9a show enhanced apoptosis at 48 hours after AraC treatment (Figures 3E and 6E), it is likely that other mechanisms, including senescence, cell cycle arrest, and autophagy, may contribute to the differential response of leukemia cells expressing MLL-AF9 or AML1-ETO9a to AraC treatment.

The leukemia stem cells in AML1-ETO-induced leukemia are HSCs whereas LSCs in MLL-AF9-induced leukemia are GMPs [13, 46–47]. While Necdin is highly expressed in HSCs, the expression of Necdin in progenitor and mature cells is very low [28, 48]. The reason why Necdin plays very different roles in different types of leukemia may be due to Necdin levels are different between stem cells and progenitor cells. MLL-AF9 and AML1-ETO regulates different gene expression signatures to drive leukemia development [12–13, and 47]. In addition, MLL-AF9 and AML1-ETO interact with distinct proteins in hematopoietic stem and progenitor cells [11–12, 19 and 49]. Thus, it is likely that Necdin plays a context dependent role in modulating leukemia-initiating cell quiescence and chemo-sensitivity. The mechanisms by which Necdin modulates leukemia development in response to different oncogenes require further investigation.

In summary, we discovered that Necdin null LICs expressing MLL-AF9 are less quiescent than wild-type LICs and Necdin-deficiency enhanced the response of MLL-AF9^+^ leukemia cells to chemotherapy treatment. Given that human leukemia patients with MLL-AF9 are resistant to chemotherapy [1, 11], our findings suggest that pharmacological inhibition of Necdin may hold potential as a novel therapy for leukemia patients with MLL translocations.

## MATERIALS AND METHODS

### Mice

The generation of *Necdin* null mice (C57BL/6, CD45.2) was described previously [42]. Fetal liver cells were isolated from E14.5 embryos as previously described [21]. Wild type C57BL/6 (CD45.2) and B6.SJL (CD45.1) mice were purchased from Jackson Laboratories. All mice were maintained in the Indiana University School of Medicine Animal Facility according to IACUC-approved protocols, and kept in Thorensten units with filtered germ-free air.

### Transduce fetal liver cells with retrovirus

Retroviral particles were produced by transfection of Phoenix E cells with the MSCV-MLL-AF9- IRES-GFP or MSCV-AML1-ETO9a-IRES-GFP plasmids, according to standard protocols [17]. Murine fetal liver cells were transduced on retronectin (Takara)-coated non-tissue culture plates with high-titer retroviral suspensions. Seventy-two hours after infection, GFP-positive cells were sorted by FACS.

### Flow cytometry

Flow cytometry analysis of hematopoietic stem and progenitor cells was performed as described previously [20–21]. Nuclear staining of Ki67 was done by using an FITC-anti-human Ki67 antibody (BD PharMingen) and fixation and permeabilization solutions from BD Biosciences [21]. Flow antibodies were purchased from Biolegend, eBioscience or BD Bioscience. Experiments were performed on FACSAria and FACSLSR II cytometers (BD Biosciences) and analyzed by using the FlowJo Version 9.3.3 software (TreeStar).

### Stem and progenitor cell assays

Clonogenic progenitors were determined in methylcellulose medium (MethoCult GF M3434, StemCell Technologies) using 2 × 10^3^ fetal liver cells per well (6-well plate) [20–21]. Colonies were scored after 7 days of the initial culture, and all cells were collected and washed twice in phosphate-buffered saline. Subsequently cells were cultured at 2 ×10^4^ per well in the same medium. Colony scoring and replating were repeated every 7 days for at least four times, or until no colonies were observed in the cultures.

### Transplantation assay

We transplanted 1 × 10^5^ fetal liver cells from wild type and Necdin null mice (CD45.2^+^) that expressing MLL-AF9 or AML1-ETO9a into lethally irradiated B6.SJL mice (CD45.1^+^) together with 1 × 10^5^ normal competitor cells (CD45.1^+^). The presence of GFP^+^ cells in the peripheral blood was measured by flow cytometry analysis every 4 weeks. 16 weeks following primary transplantation, we harvested bone marrow cells from mice reconstituted with wild type or Necdin null fetal liver cells and transplanted 3 × 10^6^ bone marrow cells into lethally irradiated B6.SJL mice (CD45.1^+^).

### Chemotherapy treatment

Leukemia cells expressing MLL-AF9 or AML1-ETO9a we treated with DMSO or different concentrations of cytarabine (AraC) for 24 hours, 48 hours and 72 hours, respectively [50]. The proliferation of leukemia cells was determined by FACS analysis. The viability of leukemia cells following AraC treatment was evaluated by PI/Annexin V staining. Early apoptotic cells were defined as Annexin V^+^ PI^−^ cells and late apoptotic cells were defined as Annexin V^+^ PI^+^ cells.

### Statistical analysis

The animal sample size was based on previous studies evaluating the roles of AML1-ETO9a in leukemia and POWER analysis [17]. Gehan-Breslow-Wilcoxon test was used for Kaplan-Meier survival curves. The other data were analyzed by paired or unpaired t test using GraphPad Prizm 6. ^*^, p<0.05; ^**^, p<0.01; ^***^, p<0.001; ns, not significant. All experiments were repeated at least once.
